# Transforming growth factor-beta inhibits aromatase gene transcription in human trophoblast cells via the Smad2 signaling pathway

**DOI:** 10.1186/1477-7827-7-146

**Published:** 2009-12-09

**Authors:** Hong Zhou, Guodong Fu, Hui Yu, Chun Peng

**Affiliations:** 1 Department of Biology, York University, Toronto, Ontario, M3J 1P3, Canada; 2 School of Life Science and Technology, University of Electronic Science and Technology of China, Chengdu, PR China

## Abstract

**Background:**

Transforming growth factor-beta (TGF-beta) is known to exert multiple regulatory functions in the human placenta, including inhibition of estrodial production. We have previously reported that TGF-beta1 decreased aromatase mRNA levels in human trophoblast cells. The objective of this study was to investigate the molecular mechanisms underlying the regulatory effect of TGF-beta1 on aromatase expression.

**Methods:**

To determine if TGF-beta regulates aromatase gene transcription, several reporter constructs containing different lengths of the placental specific promoter of the human aromatase gene were generated. JEG-3 cells were transiently transfected with a promoter construct and treated with or without TGF-beta1. The promoter activity was measured by luciferase assays. To examine the downstream signaling molecule mediating the effect of TGF-beta on aromatase transcription, cells were transiently transfected with dominant negative mutants of TGF-beta type II (TbetaRII) and type I receptor (ALK5) receptors before TGF-beta treatment. Smad2 activation was assessed by measuring phophorylated Smad2 protein levels in cytosolic and nuclear fractions. Smad2 expression was silenced using a siRNA expression construct. Finally, aromatase mRNA half-life was determined by treating cells with actinomycin D together with TGF-beta1 and measuring aromatase mRNA levels at various time points after treatment.

**Results and Discussion:**

TGF-beta1 inhibited the aromatase promoter activity in a time- and dose-dependent manner. Deletion analysis suggests that the TGF-β1 response element resides between -422 and -117 nucleotides upstream from the transcription start site where a Smad binding element was found. The inhibitory effect of TGF-beta1 was blocked by dominant negative mutants of TbetaRII and ALK5. TGF-beta1 treatment induced Smad2 phosphorylation and translocation into the nucleus. On the other hand, knockdown of Smad2 expression reversed the inhibitory effect of TGF-beta1 on aroamtase transcription. Furthermore, TGF-beta1 accelerated the degradation of aromatase mRNA.

**Conclusion:**

Our results demonstrate that TGF-beta1 exerts regulatory effects on aromatase gene at both transcriptional and post-transcriptional levels. The transcriptional regulation of aromatase gene by TGF-beta1 is mediated by the canonical TGF-beta pathway involving TbetaRII, ALK5 and Smad2. These findings further support the role of TGF-beta1 in regulating human placental functions and pregnancy.

## Background

Transforming growth factor-β (TGF-β) regulates many physiological processes, including reproduction [1-3]. During human pregnancy, TGF-β regulates placental trophoblast cell proliferation and differentiation, as well as hormone production [2,4-8]. TGF-β signaling is initiated at the cell surface by interaction of the ligand with receptor complexes that are composed of type I and type II receptor serine/threonine protein kinases [9]. In general, TGF-β interacts with its specific type II receptor (TβRII) and a type I receptor referred to as activin receptor-like kinase 5 (ALK5) [9-11]. ALK5 activates Smad2 and Smad3 through phosphorylation [9-11]. Following activation, Smad2 and Smad3 form complexes with a common Smad (Smad4) and enter the nucleus where they interact with other transcription factors, coactivators and corepressors to regulate gene transcription [12-14].

Aromatase, encoded by the *CYP19 *gene, is a key enzyme involved in estrogen biosynthesis [15]. The *CYP19 *gene has 9 coding exons (exon II-X) and the 5' untranslated region is encoded by exon I which is alternatively used by different tissues [15]. The gene uses multiple promoters in a tissue-specific manner, resulting in a tissue-specific regulation of the aromatase activity [16]. Although aromatase transcripts in different tissues have their own unique Exon I, they are spliced onto a common site upstream of the translation initiation site in exon II, thus resulting in the identical aromatase protein [17]. TGF-β has been found to regulate human aromatase expression in a tissue-specific manner. It decreased aromatase mRNA levels and activity in trophoblast cells [18], fetal hepatocytes [19], adipose stromal cells [5,20] and skin fibroflasts [21]. However, in osteoblast-like cells and THP-1 cells, TGF-β1 has been found to stimulate aromatase gene transcription [22]. In a leukaemic cell line FLG29.1, TGF-β1 stimulated aromatase expression and enzyme activity [23].

We have previously reported that TGF-β1 decreased aromatase mRNA levels in trophoblast cells [5]. To determine the mechanisms underlying this action, we examined the 5' flanking region of the placental specific exon I.1 of the aromatase gene and identified several Smad binding elements. We therefore proposed that TGF-β acts through the Smad pathway to inhibit aromatase transcription. Since a decrease in mRNA level may also be resulted from a decrease in mRNA stability, we also investigated whether TGF-β1 regulates aromatase mRNA stability.

## Methods

### Cell culture

JEG-3 cells were purchased from American Type Culture Collection (Rockville, MD). The cells were cultured in minimal essential medium (MEM, Canadian Life Technologies, Inc.) containing 10% fetal bovine serum (FBS, Sigma-Aldrich Canada Ltd, Oakville, ON) and antibiotics (100 IU/m penicillin, and 100 μg/ml streptomycin, purchased from Invitrogen Canada Inc. Burlington, ON).

### Expression constructs

Expression constructs for constitutively active and dominant negative ALK5, and dominant negative TβRII were kindly provided by Dr. L. Attisano (Univ of Toronto). Luciferase reporter constructs were generated using pGL3 basic luciferase reporter vector (Promega, Madison, WI). To obtain DNA fragments containing different lengths of the exon I.1 5' flanking sequence (+120 to -2538, +120 to -1333, +120 to -714, +120 to -422, and +120 to -117), PCR was performed using genomic DNA extracted from JEG-3 cells as the template. All PCRs were carried out for 30 cycles using a common antisense primer, 5'-AGATTAAGAGATACATACGCG-3', and a specific sense primers (5'-CCCGGTACCCCAGATGATCTTTCCCAGGAA-3' for Arom -2538 construct; 5'-CTGTGGAACCATGAGCCAATT-3' for Arom-1333; 5'-GCATTGGAGATACGGAAGTAA-3' for Arom -714; 5'-TGTAGAACAATGTGGTGTGTG-3' for Arom-422; and 5'-AGACCTTGCTGAGATTAGATC-3' for Arom-117). The resulting DNA fragments were first cloned into pCRII vector using a TOPO cloning kit (Invitrogen). The inserts were then cut out from pCRII vector using KpnI and XhoI (for Arom -1333, Arom -714 bp, Arom -422 bp and Arom -117 bp) and KpnI and NheI (for Arom -2541) respectively, and then subcloned into the pGL3-basic vector. All promoter constructs were fully sequenced to verify their identities.

### Transient transfection and luciferase assay

JEG-3 cells were seeded into 6-well plates at a density of 2 × 10^5 ^cells/well and incubated in MEM containing 10%FBS for 24 hours. Transient transfection was carried out using a 25 kDa polyethylenimine (PEI, Sigma-Aldrich) as previously described [24]. After transfection for 4 h, the medium was replaced with MEM supplied with 10% FBS and cells were allowed to be recovered for different lengths of time. After treatment with TGF-β1, cells were then lysed in 250 μl of lysis buffer (20 mM Tris PH 7.4/0.1% TritonX-100). The supernatant was collected for both luciferase and β-gal assays. To determine the luciferase activity, 100 μl of luciferase substrate (Promega) was added to 30 μl supernatant and the total light emission during the initial 10 seconds of the reaction was measured using a luminometer. For β-gal assay, 30 μl of supernatant was added into 270 μl of reaction mixture (1 ml contains 244 μl of 4 mg/ml of 2-Nitrophenyl-β-D-Galactopyranoside (ONPG), 2.22 μl of 0.5 M MgSO_4_, 3.89 μl of β-mercaptoethanol, 186.2 μl of 0.4 M sodium phosphate buffer, and 563.9 μl of water, and all chemicals were purchased from Sigma-Aldrich), mixed well and incubated at 37°C for 30 min or until a yellow color change was detectable. The reaction was then terminated by the addition of 500 μl of 1 M Na_2_CO_3 _and absorbance at 410 nm was measured using a spectrophotomer. The luciferase assay data were normalized by β-gal activity and expressed as relative luciferase activity.

### Treatment with TGF-β1

Cells were plated into 6-well plates at the density of 2 × 10^5^cells/well and allowed to grow overnight. To determine the effect of TGF-β1 on aromatase gene transcription, cells were either treated with different concentrations of TGF-β1 for 2 hours or 1 ng/ml of TGF-β1 for different time periods (2 h, 6 h, 12 h and 24 h).

### Silencing of Smad2 expression

Smad2 expression was knockdown using its siRNA. The published Smad2 siRNA sequence (UCUUUGUGCAGAGCCCCAAtt [25]) was cloned into the pSuper vector (Oligoengine, Seattle, WA), and JEG-3 cells were transiently transfected with either the control pSuper vector or the siRNA for Smad2 for 4 h. After recovering for 12, 24, 48 and 72 h, protein extract was prepared from cells and Western blotting was performed using an antibody against human Smad2 (Cell Signalling). To determine the effect of Smad2 silencing on TGF-β action, cells were first transfected with the control or siRNA plasmid for 4 h and recovered for 24 h before being treated with TGF-β1 (1 ng/ml) for 6 h.

### Protein extraction and Western blot analysis

The whole cell extraction was carried out as described previously with some modification [26]. Briefly, JEG-3 cells were plated using a density of 6 × 10^5 ^cells/dish in 60-mm dishes and incubated overnight. Following treatment with 1 ng/ml TGF-β1 for 30 min, the cells were lysed in lysis buffer (50 mM Tris-HCl, 150 mM NaCl, 1% Triton X-100, 0.5% deoxycholate, and 1% SDS) containing 1 mM dithiothreitol, 1 mM Na_3_VO_4_, 5 mM NaF, 100 mM EDTA, 10 mg/ml aprotinin, and 100 mM phenylmethysulfonyl fluoride. Nuclear proteins and cytoplasm proteins were extracted using Nuclear Extraction Kit (Panomics) following the manufacturer's protocols and stored at -20°C until Western blot analysis. Proteins (40 μg) were separated by SDS-polyacrylamide gel electrophoresis in 12% gels. The proteins were transferred electrophoretically onto polyvinylidene difluoride membranes (Immobilon-P, Millipore Corp., Bedford, MA). After blocking with 5% milk in TBS-T, the membranes were incubated with rabbit anti-human phospho-Smad2 (1:1000 dilution, Cell Signaling) or phosphor-Smad3 (1:1000 dilution, Cell Signaling), or mouse anti-human actin (1:1000 dilution, Sigma) antibodies at 4°C overnight. The membranes were subsequently probed with horseradish peroxidase-conjugated anti-rabbit or anti-mouse (1:5000 dilution, Amersham) at room temperature for 1 h. Signals were detected using a ECL-Plus kit (Amersham) according to the instructions of the manufacturer.

### Aromatase mRNA stability assays

To study the effect of TGF-β1 on aromatase mRNA stability, JEG-3 cells were seeded at a density of 1 × 10^6 ^cells/60 mm dish. 24 h after plating, cells were treated with 2 μg/ml actinomycin D (Sigma), either alone or in the presence of 1 ng/ml of TGF-β1, in serum free medium. The cells were then harvested at time 0 (pretreatment) and 6, 12, 24, 36 and 48 h after treatment with TGF-β1. Total RNA was then extracted using Trizol reagent (Invitrogen) following the manufacturer's recommended protocol and reversed transcribed into cDNA as previously described [5]. Aromatase mRNA levels were determined using RT-PCR. GAPDH was used as internal control to normalize the aromatase mRNA level. The GAPDH sense primer was 5'-AAGGTCATCCCTGAGCTGAAC, and antisense primer was 5'-CGCCTGCTTCACCACCTTCTA. Primers used in aromatase PCR were sense: 5'-GCTGCAGTGCATCGGTATGCA-3', and antisense: 5'-ACTCGAGTCTGTGCATCCTTC-3'. Validation assays were performed to determine the proper cycle number and the numbers chosen for aromatase (32 cycles) and GAPDH (22 cycles) are in the linear range of amplification.

### Statistical analysis

All transfection assays were performed in triplicate and repeated at least three times. The relative luciferase activity shown in figures was the Mean ± SEM for triplicate wells from one representative experiment. Statistical analysis was done with one-way ANOOVA, followed by a Student-Newman-Keul's multiple comparison tests using Graph Pad InStat software (Graph Pad Inc., San Diego, CA). For comparison between two groups, Student's *t *test was used. Differences were considered statistically significant at P < 0.05.

## Results

### TGF-β1 inhibits aromatase gene transcription

**Figure 1:**
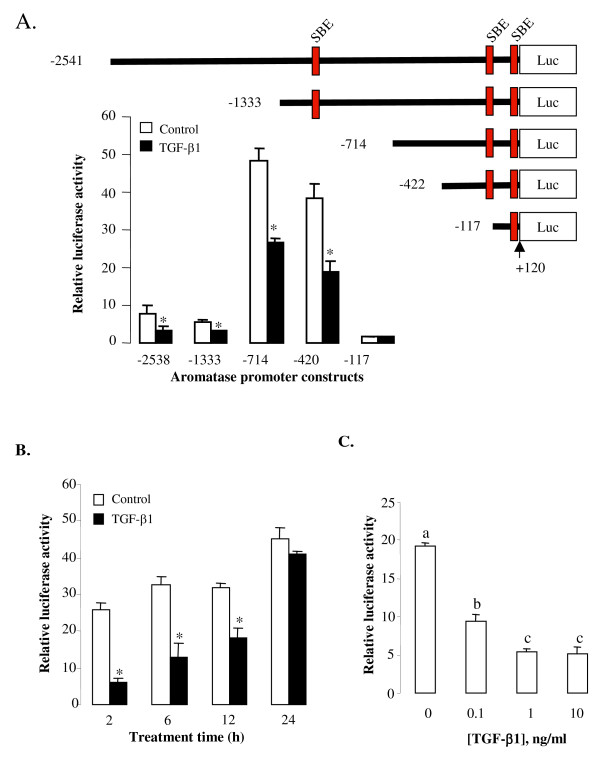
**Placental aromatase promoter constructs and the effect of TGF-β1 on promoter activities**. A) Various lengths (-2538 to -117) of the 5' flanking region of the placental specific exon 1 were cloned into a luciferase reporter construct pGL3 basic. Smad binding elements (SBE) are shown at the red box region. JEG-3 cells cultured in 6-well plates were transfected with these luciferase constructs (1 μg) and then treated with or without TGF-β1 (1 ng/ml) for 6 h. B) Cells were transfected with Arom -714 construct and then treated with TGF-β1 (1 ng/ml) for the duration as indicated. C) Cells were transfected with Arom -714 - and then treated with different concentrations of TGF-β1 for 6 h. In these experiments, a β-galactosidase expression vector (0.5 μg) was co-transfected into cells for normalizing transfection efficiency. Relative luciferase activity was calculated as the ratio of luciferase/β-gal. Data are mean ± SEM of three replicates from one experiment. The experiment has been repeated twice with similar results. *, P < 0.05 verse empty vector control.

Examination of the major promoter region that directs placenta-specific expression of the aromatase gene reveals the presence of four Smad binding elements (SBE) [27], CAGAC, located at +93 to +97, -114 to -118, -118 to -122, and -1186 to -1182 (Fig. 1A). This suggests that the TGF-β family may regulate aromatase transcription via the Smad pathway. Since TGF-β1 decreased aromatase mRNA [5], we therefore tested its effect on aromatase gene transcription. Five promoter constructs containing different lengths of the 5' flanking sequence of the exon I.1, designated as Arom -2538, Arom -1333, Arom -714, Arom -422, and Arom -117, were made and used to test the effect of TGF-β1. JEG-3 cells were transiently transfected with a luciferase construct and then treated with or without TGF-β1. TGF-β1 inhibited promoter activities in Arom -2538, Arom -1333, Arom -714, and Arom -422 constructs. Deletion between -422 and -117 abolished the basal promoter activity as well as the inhibitory effect of TGF-β1 (Fig. 1A).

The dose-dependent and time-course effects of TGF-β1 were subsequently tested using promoter construct Arom -714. A significant decrease in aromatase promoter activity was observed at 2 to 12 h after TGF-β1 treatment (Fig. 1B). At 6 h after treatment, all doses of TGF-β1 (0.1 to 10 ng/ml) significantly inhibited luciferase activity; with a maximal effective dose observed at 1 ng/ml (Fig. 1C).

### TGF-β1 acts through TβRII, ALK5, and Smad2 to regulate aromatase gene transcription

**Figure 2:**
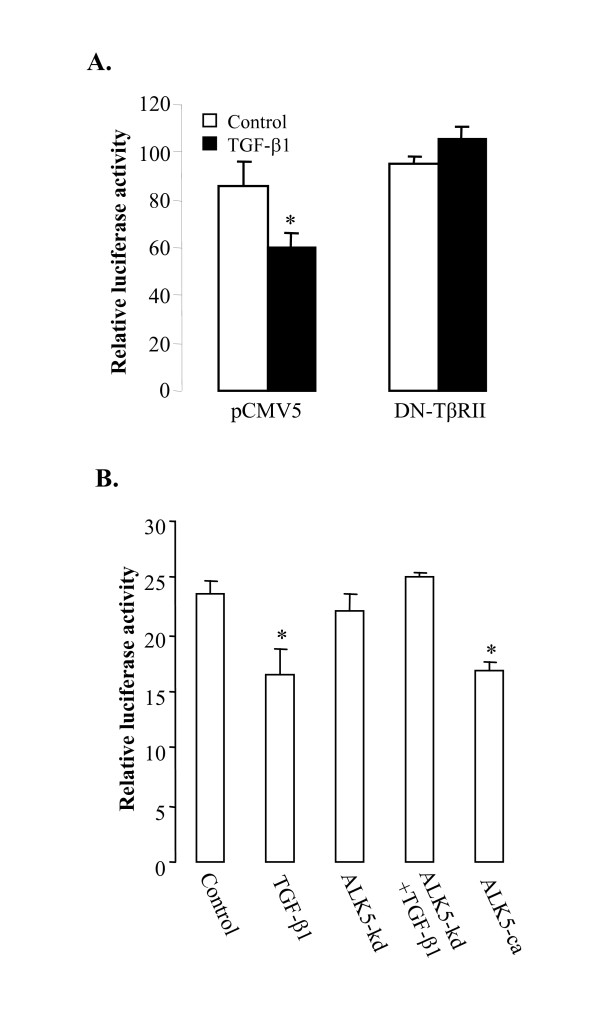
**TGF-β1 acts through TβRII and ALK5 to inhibit aromatase transcription**. A) Cells were transfected with Arom -714 construct, the control vector pCMV5 or dominant negative TβRII. At 24 h after transfection, they were treated with the control medium or TGF-β1 (1 ng/ml). B) Cells were transfected with Arom -714 construct, control vector pCMV5, the dominant negative ALK5 (ALK5-kd) and the constitutively active ALK5 (ALK5-ca). A β-galactosidase expression vector (0.5 μg) was simultaneously co-transfected into cells for normalizing transfection efficiency.

To confirm that TGF-β1 acts through its specific receptors and the Smad pathway to regulate aromatase gene transcription, several experiments were performed. First, cells were transfected with dominant negative mutant of TβRII, or its vector control (pCMV5) and then treated with or without 1 ng/ml TGF-β1. As shown in Fig. 2A, TGF-β1 treatment decreased luciferase activity in the pCMV5-transfected cells, but not in the dominant negative TβRII-transfected cells, indicating that dominant negative TβRII reversed the inhibitory effect of TGF-β1 on aromatase gene transcription. Second, the involvement of ALK5 in TGF-β1-regulated aromatase transcription was examined using both constitutive active and dominant negative mutants. Transfection of the constitutively active ALK5 inhibited the aromatase promoter activity to a similar extend as TGF-β1 treatment (Fig. 2B). On the other hand, overexpression of dominant negative ALK5 completely blocked the effect of TGF-β1 on aromatase promoter activity (Fig. 2B).

**Figure 3:**
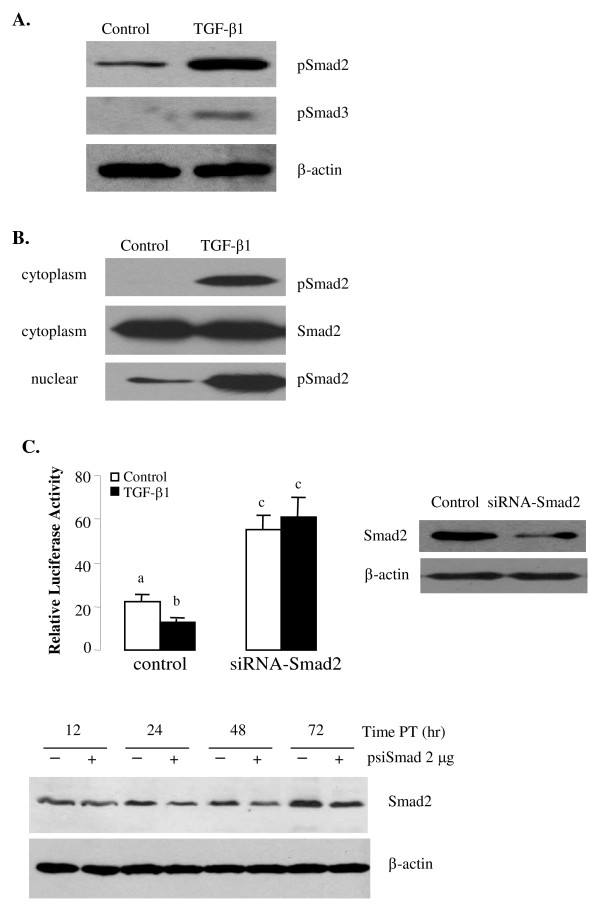
**Smad2 is involved in TGF-β1-inhibited aromatase transcription**. A) TGF-β1 induced Smad2 phosphorylation. Cells were treated with TGF-β1 (1 ng/ml) for 1 h and Smad2 and Smad3 phosphorylation was determined by Western blot analysis using specific antibodies. β-actin was used for the loading control. B) TGF-β1 induced Smad2 nuclear translocation. Cells were treated with TGF-β1 as above and cytoplamic and nuclear proteins were analyzed for the phosphorylated Smad2 levels. C) Validation of Smad2 siRNA. Cells were transfected with 2 μg of pSuper empty vector or Smad2 siRNA (siSmad2) cloned in pSuper. At various time points after transfection, cell lysates were prepared and probed for Smad2. D) Smad2 siRNA reversed the TGF-β1-inhibited aromatase transcription. Cells were transfected with pSuper or pSuper carrying Smad2 siRNA. Smad2 siRNA blocked the effect of TGF-β1 on aromatase transcription. Knockdown of Smad2 by its siRNA was confirmed by Western blotting.

We subsequently investigated if the Smad pathway is involved in the TGF-β1-regulated aromatase transcription. First, protein samples were extracted from cells at 30 min after TGF-β1 treatment and immunoblotting was performed. TGF-β1 strongly induced Smad2 phosphorylation (Fig. 3A). Consistent with our previous report that Smad3 expression level is very low in JEG3 cells [28], pSmad3 was only weakly detected in the TGF-β1-treated cells (Fig. 3A). To further confirm the activation of Smad2, protein samples were prepared from cytoplasmic and nuclear fractions of the control and TGF-β1-treated cells. Treatment with TGF-β1 resulted in strong accumulation of phosphorylated Smad2 in the nucleus (Fig. 3B). To directly examine the role of endogenous Smad2, we generated a siRNA construct to silence Smad2 expression. Compared to the vector control, transfection with siSmad2 construct resulted in a decrease in Smad2 protein levels at 24, 48 and 72 h after transfection (Fig. 3C). When cells were transfected with siSmad2 construct before TGF-β1 treatment, we found that knockdown of Smad2 completely reversed the effect of TGF-β1 on aromatase transcription (Fig. 3D).

### TGF-β1 decreased aromatase mRNA stability

**Figure 4:**
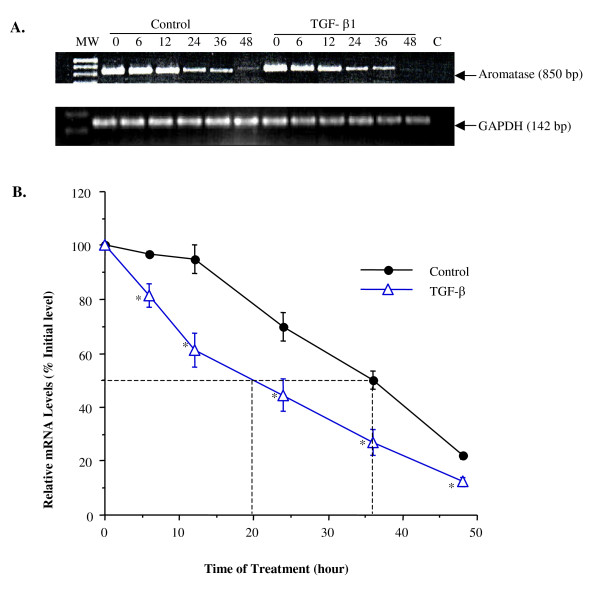
**TGF-β1 decreased aromatase mRNA stability**. Cells seated in 24-well plates were treated with 2 μg/ml actinomycin D alone or in combination with TGF-β1 (1 ng/ml). Cells were harvested at time 0 (pretreatment), 6, 12, 24, 36 and 48 h after treatment with TGF-β1. Aromatase mRNA levels were determined by semi-quantitative PCR. A) Representative results from one experiment. B) The half-life (T_1/2_) of aromatase transcript is defined as the time required for aromatse mRNA levels to drop to 50% of its starting values (0 time point). In this study, the T_1/2 _value of aromatse mRNA was reduced from 36 h in the control to 20 h in the treatment groups with TGF-β1. Data are mean ± SEM of three experiments. *, P < 0.05 verse the time matched control.

Since the decrease in aromatase mRNA levels could also be due to a decrease in its stability, we tested if TGF-β1 regulated aromatase mRNA degradation. Cells were treated with a transcription inhibitor actinomycin D and the decay of aromatase mRNA was measured from 6 to 48 h. At all time points tested, aromatase mRNA levels were significantly lower in TGF-β1-treated cells than in the control cells (Fig. 4). The half-life of aromatase transcript was approximately 36 h and 20 h, respectively, in the control and TGF-β1-treated cells (Fig. 4B), suggesting that TGF-β1 decreased the half-life of aromatase mRNA by 16 h.

## Discussion

Aromatase plays important roles during pregnancy as it is the key enzyme involved in estrogen production. TGF-β has been reported to regulate placental development and functions, including estrogen synthesis. In this study, we demonstrated that TGF-β1 inhibits aromatase transcription and decreases aromatase mRNA stability, thus providing new insights into the molecular mechanisms underlying the effect of TGF-β1 in placental steroidogenesis.

Using luciferase reporter assays, we found that TGF-β1 inhibited promoter activity of aromatase gene. Deletion analysis showed that removal of nucleotides between -422 to -117 resulted in a loss of response to TGF-β1, as well as the basal promoter activity. This finding suggests that the TGF-β response element resides in the region between -117 and -422. Since there are two overlapping SBEs located at -114 to -118 and -118 to -122, it is possible that these SBEs are important in mediating the effect of TGF-β1 on aromatase transcription. Additional SBEs found in +93 to +97 and -1186 to -1182 may not participate in TGF-β-regulated aromatase transcription. Our finding that -117 to -422 region is important for maintaining the basal promoter activity is consistent with previous studies which showed that constructs containing up to -125 bp of the placenta aromatase promoter had no transcriptional activity [29,30].

TGF-β signals through a receptor complex containing type I and type II serine/threonine kinases. It binds to type II receptors which in turn recruit and phosphorylate type I receptors, resulting the activation of type I receptors. Several type I receptors have been reported to mediate TGF-β signaling, including ALK1 and ALK5 [11]. While ALK5 is expressed in a variety of cells, ALK1 expression is restricted to endothelial cells [11]. In this study, we found that the dominant negative mutant of TβRII and ALK5 completely reversed the inhibitory effect of TGF-β1 on aromatase transcription while constitutively ALK5 mimicked the effect of TGF-β1. These results indicate that TGF-β1 acts through TβRII and ALK5 to regulate aromatase expression. ALK5 is known to activate the Smad2/3 pathway [9,11]. Consistent with this, we found that TGF-β1 induced Smad2 phosphorylation and nuclear accumulation in JEG3 cells. Silencing of Smad2 expression using siRNA resulted in the loss of TGF-β1 inhibitory effect on aromatase transcription, demonstrating that Smad2 mediates the effect of TGF-β1 on these cells. TGF-β1 also activated Smad3; however, since Smad3 expression is very low in JEG3 cells, it is unlikely that it plays a role in TGF-β1-regulated aromatase promoter activity.

In addition to regulating aromatase transcription, TGF-β1 also decreased aromatase mRNA stability since we found that in the presence of actinomycin D, TGF-β1 was able to reduce aromatase mRNA levels by shortening its half-life. The mechanism whereby TGF-β1 reduces aromatase mRNA stability is unclear at present. Recently, it was reported that the tissue specific alternative exon I of the aromatase gene play an important role in the posttranscriptional regulation of aromatase gene expression [31]. Whether or not the TGF-β1 pathway can target the placental specific exon I.1 to regulate aromatase mRNA stability remains to be determined.

## Conclusion

**Figure 5:**
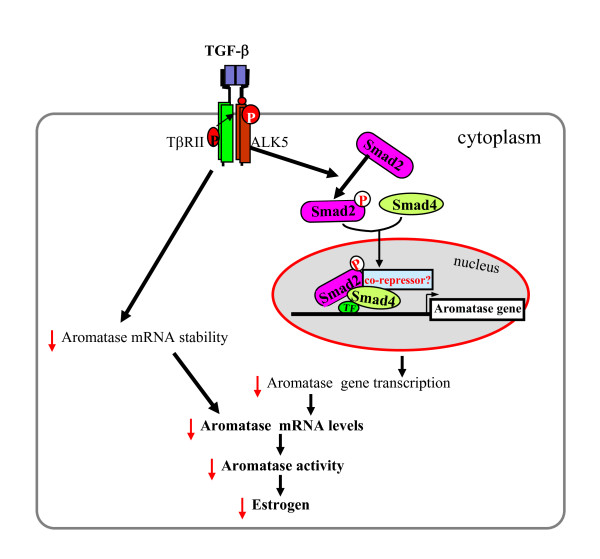
**Proposed model of TGF-β action in estrogen production in trophoblast cells**. TGF-β binds to its serine/threonine receptor complex (TβRII and ALK5) to activate Smad2. The phosphorylated Smad2 forms a complex with Smad4 and translocate to the nucleus, where the complex could interact with other transcription factors (TF) and co-repressors to inhibit aromatase gene transcription. TGF-β also decreases aromatase mRNA stability, which contributes to a decrease in aromatase mRNA levels. The decrease in aromatase mRNA may lead to a decrease in aromatase activity and thus estrogen biosynthesis in trophoblast cells.

In summary, we have shown that TGF-β1 exerts its regulatory effects on aroamtase mRNA expression at both transcriptional and post-transcriptional levels. TGF-β1 acts through the canonical TGF-β signaling pathway involving TβRII, ALK5, and Smad2 to inhibit aromatase transcription and through unknown mechanisms to decrease aroamtase mRNA stability. This could in turn decrease aromatase activity, leading to inhibition of estrogen biosynthesis (Fig. 5). These findings further support the role of TGF-β1 in regulating human placental functions and pregnancy.

## Competing interests

The authors declare that they have no competing interests.

## Authors' contributions

HZ carried out the aromatase promoter studies and participated in the design of the study and in manuscript writing. GF conducted the Smad2 siRNA study, repeated many of the luciferase assays and participated in drafting the manuscript. HY performed the aromatase mRNA stability study. CP conceived of the study, participated in its design, and drafted the manuscript. All authors read and approved the final manuscript.
